# Outcomes of Noninvasive Positive Pressure Ventilation in Acute Respiratory Distress Syndrome and Their Predictors: A National Cohort

**DOI:** 10.1155/2019/8106145

**Published:** 2019-09-18

**Authors:** Ahmed Taha, Eneko Larumbe-Zabala, Ashraf Abugroun, Assad Mohammedzein, M. Tarek Naguib, Manish Patel

**Affiliations:** ^1^Department of Internal Medicine, Texas Tech University Health Sciences Center, Amarillo, TX, USA; ^2^Clinical Research Institute, Texas Tech University Health Sciences Center, Lubbock, TX, USA; ^3^Department of Internal Medicine, Advocate Illinois Masonic Medical Centre, Chicago, IL, USA; ^4^Department of Internal Medicine, Division of Pulmonary and Critical Care Medicine, Texas Tech University Health Sciences Center, Amarillo, TX, USA

## Abstract

**Rationale:**

Although noninvasive positive pressure ventilation (NIPPV) is increasingly used in acute respiratory distress syndrome (ARDS) to avoid invasive mechanical ventilation (IMV), the data supporting its benefit for this indication are lacking.

**Objectives:**

To analyze the all-cause in-hospital mortality rate and length of stay (LOS) for ARDS patients who received NIPPV in the United States (US) compared to those who were initially intubated. Our secondary outcome of interest was to determine the predicting factors for NIPPV failure.

**Methods:**

We used the 2016 National Inpatient Sample database to identify 4,277 adult records with ARDS who required positive pressure ventilation. We divided the cohort into initial treatment with IMV or NIPPV. Then, the NIPPV group was further subdivided into NIPPV failure or success. We defined NIPPV failure as same-patient use of NIPPV and IMV either on the same day or using IMV at a later date. We analyzed the in-hospital mortality, LOS, and NIPPV failure rate. Linear regression of log-transformed LOS and logistic regression of binary outcomes were used to test for associations.

**Results:**

The NIPPV success group had the lowest mortality rate (4.9% [3.8, 6.4]) and the shortest LOS (7 days [6.6, 7.5]). The NIPPV failure rate was 21%. Sepsis, pneumonia, and chronic liver disease were associated with higher odds of NIPPV failure (adjusted OR: 4.47, 2.65, and 2.23, respectively). There was no significant difference between NIPPV failure and IMV groups in-hospital mortality (26.9% [21.8, 32.8] vs. 25.1% [23.5, 26.9], *p*=0.885) or LOS (16 [14, 18] vs. 15.6 [15, 16.3], *p*=0.926).

**Conclusions:**

NIPPV success in ARDS exhibits significantly lower hospital mortality rates and shorter LOS compared with IMV, and NIPPV failure exhibits no significant difference in hospital mortality or LOS compared with patients who were initially intubated. Therefore, an initial trial of NIPPV may be considered in ARDS. Sepsis, pneumonia, and chronic liver disease were associated with higher odds of NIPPV failure; these factors should be used to stratify patients to the most suitable ventilation modality.

## 1. Introduction

The use of noninvasive positive pressure ventilation (NIPPV) in acute respiratory failure (ARF) is increasing primarily to avoid the adverse events of invasive mechanical ventilation (IMV) [1]. NIPPV accounts for approximately 40% of total ventilator starts for ARF and up to 80% of starts in patients with exacerbations of chronic obstructive pulmonary disease (COPD) or acute cardiogenic pulmonary edema (ACPE) [2]. Despite the tremendously improving success rate of NIPPV over the last decade in ARF regardless of the underlying etiology [3] and the presence of strong evidence supporting its use for acute COPD exacerbations and ACPE [2, 4], the evidence supporting NIPPV use over IMV in acute respiratory distress syndrome (ARDS) is lacking and controversial.

Several studies showed a universal beneficial role of NIPPV in ARDS [5], and others emphasized its role in certain ARDS populations, such as immunocompromised patients or those with mild disease [6, 7]; conversely, other studies have shown a poor track record of NIPPV use in ARDS [8, 9]. Consequently, these conflicting findings have resulted in major restrictions and concerns for NIPPV use in ARDS. Additionally, the subgroup of patients with ARDS likely to benefit from or fail the trial of NIPPV still remains unclear [10], and all previously published studies that sought to predict NIPPV failure in ARDS were not conclusive primarily due to inadequate sample size. Therefore, we aimed to conduct the largest retrospective cohort study to analyze the all-cause in-hospital mortality and length of stay (LOS) for ARDS patients who received NIPPV in the United States (US). Our secondary outcome of interest was to determine the predicting factors for NIPPV failure.

## 2. Methods

### 2.1. Study Setting

This is a retrospective cohort study that was conducted using the 2016 National Inpatient Sample (NIS) part of Healthcare Cost and Utilization Project (HCUP), which is sponsored by the Agency for Healthcare Research and Quality (AHRQ) [11]. This study includes deidentified sample data of hospital discharges from 47 states; when weighted to include nationwide discharges, this study represents more than 97 percent of the US population [12]. Each hospitalization is treated as an individual database entry, and the individual weights supplied by the AHRQ are used in all analyses to maintain the integrity of the complex survey design and allow for extrapolation of the findings to the entire US population. The Institutional Review Board at the Texas Tech University Health Sciences Center deemed this study exempt from review due to the use of deidentified data.

### 2.2. Study Design

All hospitalization records were analyzed using the International Classification of Diseases, Tenth Revision, Clinical Modification and Procedure Coding System (ICD-10 CM/PCS), consistent with the previously published literature [13, 14]. We used the principal diagnosis codes linked to each hospitalization to identify all adult records with ARDS during the 2016 calendar year, which represents the study period. We then used ICD-10 PCS codes to identify records of ARDS that required NIPPV, IMV, or both. The following data were extracted from the NIS database: patient and hospital demographics, admission and treating diagnoses, in-patient procedures, in-hospital mortality rates, hospital length of stay, and discharge status [11]. We used the Elixhauser Comorbidity Index provided by the HCUP-NIS to derive the prevalence of comorbidities in our sample [15]. Additionally, we ran ICD-10 codes to assess the prevalence of ARDS etiologies and the incidence of in-hospital complications. [Supplementary-material supplementary-material-1] in the Online Supplement provides the ICD-10 CM/PCS codes that were used to identify the dataset records.

To avoid misclassification bias and in compliance with Berlin criteria for ARDS, we excluded all records with acute respiratory failure that could have been attributed to (ACPE) and those who were on high-flow oxygen at the time of diagnosis [16]. We also excluded all records with “Do not resuscitate/Do not intubate” status due to its possible interference with the decision to intubate if it was clinically indicated. We also excluded discharges related to patients with missing time-to-procedure to improve the study precision. To further improve the precision and internal validity, records with IMV application prior to admission, such as at-the-scene intubations, were excluded from the cohort because the indication for intubation was not identifiable and might not correlate to the study diagnosis of ARDS. Moreover, we treated all observations as hospitalization events rather than unique patients [17] and avoided the use of nonspecific secondary diagnosis codes to infer in-hospital events [18]. If the NIS strata did not present records for any of the groups when the complex survey design was applied, we excluded that stratum from the final data analysis [19].

**Figure 1:**
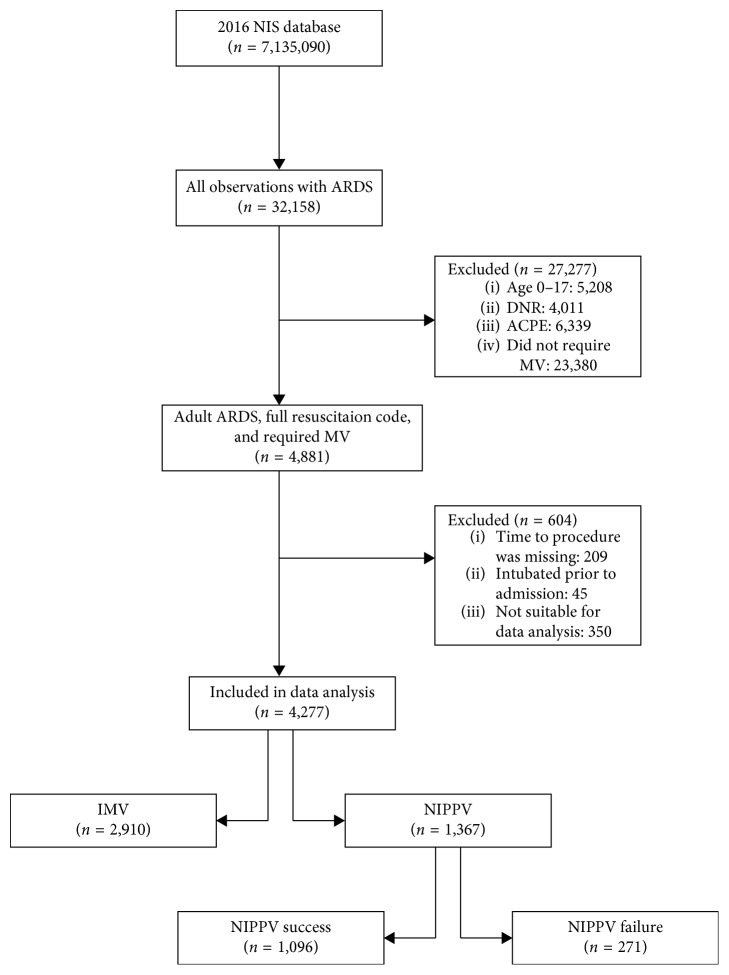
Study design flow chart. NIS: national in-patient sample; *n*: actual number of observations,; ARDS: acute respiratory distress syndrome; DNR: do not resuscitate; ACPE: acute cardiogenic pulmonary edema; MV: mechanical ventilation; IMV: invasive mechanical ventilation; NIPPV: noninvasive positive pressure ventilation.

We then divided the cohort into the IMV and NIPPV groups. The IMV group represented patients who were initially intubated regardless of whether NIPPV was used afterwards. Records with NIPPV use after IMV during the same hospitalization, i.e., as a step-down weaning method in patients at risk of postextubation CO_2_ retention, are still included in the IMV group. The NIPPV group was subdivided into the NIPPV success and failure subgroups. The NIPPV success subgroup included ARDS patients who received NIPPV treatment and were never intubated, while the NIPPV failure subgroup represented ARDS patients who received both NIPPV and IMV either on the same day or IMV at a later date than NIPPV.  Figure 1 shows the study design flow chart.

### 2.3. Statistical Analysis

Complex survey design and population weights provided in the HCUP-NIS database were used for all statistical analyses; national estimates for ARDS hospitalizations, race, and sex were generated accordingly. Demographic and clinical characteristics were summarized as the mean and percentage with 95% confidence interval (CI) for continuous and categorical variables, respectively. Simple logistic regression models were used to assess the association between in-hospital mortality as an outcome variable and the following predictors: study group, indicator of sex, age (in years) at admission, race (uniform), hospital type and location, ARDS etiologies (sepsis, pneumonia, acute pancreatitis, transfusion-related acute lung injury (TRALI), and trauma), and AHRQ comorbidity measures (chronic congestive heart failure, chronic obstructive pulmonary disease, chronic ischemic heart disease, chronic kidney disease, chronic liver disease, malignancy, dementia, hypertension, diabetes mellitus, obesity, and smoking). Data are presented as the odds ratio (OR) with a 95% CI. Statistically significant predictors were then selected and used in subsequent multiple logistic regression models to determine adjusted coefficients and presented as adjusted odds ratios (aOR) with 95% CI.

LOS presented as a nonnormal distribution; thus, it was log-transformed before running the regression models. Unadjusted and adjusted coefficients for log-LOS predictors were calculated using simple and multiple linear regression models, respectively, with the same independent variables used for mortality. After multiple regression for both mortality and log-LOS, margins for study groups were estimated and compared in a pairwise manner. Bonferroni correction was used to control multiple-comparison error. The *p* value significance level was set at 0.05.

To determine the significant predictors for NIPPV failure, simple logistic regression models were tested for demographic variables, ARDS etiologies, and comorbidities. Then, only statistically significant predictors were further analyzed to obtain the aORs using the multiple logistic regression model (see [Supplementary-material supplementary-material-1] in the Online Supplement). All data were analyzed using STATA 15.1 (StataCorp, College Station, TX).

## 3. Results

Among 7.1 million all-cause hospitalization records in the 2016 NIS database, we identified 4,277 adult records with a primary diagnosis of ARDS that fulfilled the inclusion criteria. IMV was used in the majority of the included cohort (68%). Patients who were initially intubated were slightly younger than those who were on NIPPV treatment (56.3 vs. 61.5 years) but had similar demographics and comorbidity burden otherwise. Sepsis (44.1%), pneumonia (38.7%), and trauma (9.2%) were the most prevalent ARDS etiologies in the overall study population and each individual group, while acute pancreatitis (3.2%) and TRALI (0.4%) were the least reported etiologies. Acute kidney injury (AKI) and the development of new-onset shock state were the most incident complications in the IMV (49.6% and 40.8%, respectively) and NIPPV groups (32.8% and 14%, respectively).  Table 1 shows patient and hospital characteristics, ARDS etiologies, comorbidities, and complications based on the study group.

NIPPV was initially used in 1,367 ARDS patients (32%), of whom 287 (21%) failed and were eventually intubated. The all-cause in-hospital mortality rate was 25.1% in the IMV group, 4.9% in the NIPPV success group, and 26.9% in the NIPPV failure. The mean LOS was 15.6 days (95% CI [15, 16.3]) in the IMV group, 7 days (95% CI [6.6, 7.5]) in the NIPPV success group, and 16 (95% CI [14, 18]) in NIPPV failure group.  Table 2 shows the prevalence of the outcomes based on the study group.

**Figure 2:**
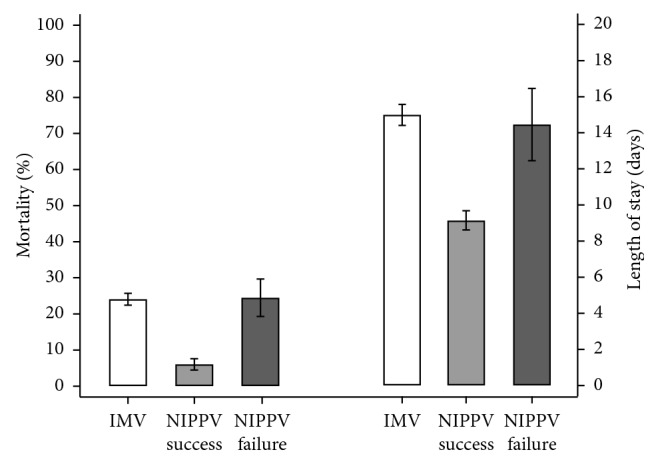
Adjusted estimates with 95% CI for all-cause in-hospital mortality (left) and length of stay (right) based on study group. IMV: invasive mechanical ventilation; NIPPV: noninvasive positive pressure ventilation.

In the multivariate logistic regression, there was no significant difference in hospital mortality between the NIPPV failure and IMV groups (26.9% [21.8, 32.8] vs. 25.1% [23.5, 26.9], *p*=0.885). Similarly, the adjusted model for log-transformed LOS (see [Supplementary-material supplementary-material-1] in the Online Supplement) showed no significant difference between the IMV and NIPPV failure groups for LOS (16 [14, 18] vs. 15.6 [15, 16.3], *p*=0.926). However, the NIPPV success group had significantly lower all-cause in-hospital mortality rates; both the IMV (aOR 5.3 [3.96, 7.11]) and NIPPV failure (aOR 5.43 [3.61, 8.17]) groups showed higher odds of hospital morality compared with the NIPPV success group (see [Supplementary-material supplementary-material-1] in the Online Supplement). The NIPPV success group also showed significantly shorter LOS (7 days [6.6, 7.5]) compared with the IMV (*p* < 0.001) and NIPPV failure (*p* < 0.001) groups.  Figure 2 shows the adjusted estimates and 95% CI for the outcomes based on study groups.

**Figure 3:**
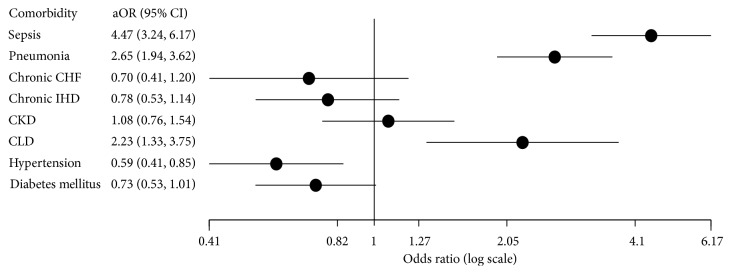
Adjusted odds ratios of noninvasive positive pressure ventilation failure for associated comorbidities. aOR: adjusted odds ratio; CI: confidence interval; CHF: congestive heart failure; IHD: ischemic heart disease; CKD: chronic kidney disease; CLD: chronic liver disease.

After adjusting for other significant factors, sepsis, pneumonia, and chronic liver disease were found to be associated with higher odds for NIPPV failure. Sepsis exhibited a large effect size (aOR 4.47 [3.24–6.17]), while pneumonia and liver disease showed a moderate effect size (aOR 2.65 [1.94–3.62] and 2.23 [1.11–3.75], respectively).  Figure 3 shows adjusted estimates and 95% CI for all factors associated with NIPPV failure.

## 4. Discussion

The Berlin definition of ARDS requires that all of the following criteria be present for diagnosis: respiratory symptoms within one week of a known clinical insult; bilateral opacities in chest imaging that are not otherwise fully explained; respiratory failure is not fully explained by ACPE; and impairment of oxygenation on ventilator settings that include positive end-expiratory pressure (PEEP) or continuous positive airway pressure (CPAP) ≥5 cm H_2_O [20].

Most clinicians tend to use IMV for ARDS patients liberally and reserve the use of NIPPV for patients with ARDS who are hemodynamically stable, have milder disease, are easily oxygenated, and have no contraindications to its use. This conservative approach is based on conflicting data regarding the benefits and harm of NIPPV in the ARDS population [5, 8]. For example, a study of patients with acute hypoxemic respiratory failure reported increased mortality in association with NIPPV compared with high-flow nasal cannula. In this study, a low tidal volume was almost impossible to achieve in most patients receiving NIPPV, and a high tidal volume was independently associated with NIPPV failure. Nonetheless, whether the potential harm associated with NIPPV reported in this study was due to the delivery of higher than expected tidal volumes remains unclear [21].

Additionally, an analysis from the Large Observational Study to Understand the Global Impact of Severe Acute Respiratory Failure (LUNG-SAFE) reported an increase in intensive care unit but not hospital mortality with the use of NIPPV in ARDS patients who have severe hypoxemia (PaO_2_/FiO_2_ ratio <150) and that clinicians should defer the use of NIPPV in such patients [7]. These conclusions should be interpreted with caution because the results of the LUNG-SAFE study were partly discrepant with the propensity-matched analysis due to low study power and the smaller number of patients included. It is also worth mentioning that the NIPPV failure rate in the LUNG-SAFE study was underreported because patients treated with NIPPV on day 1 were excluded [8].

On the other hand, several other studies supported the use of NIPPV in ARDS, especially in its milder forms. For instance, helmet-delivered NIPPV reduced the need for intubation in mild or moderate ARDS. It was also associated with a higher rate of ventilator-free days, shorter ICU stay, and lower 90-day mortality without an increase in adverse effects [22]. Although it provided promising results, this study was small, single-center, unblinded and it was stopped early, which may make it more likely that the effect size is exaggerated. Another multiple-center survey on the use of NIPPV as a first-line intervention for ARDS has shown that intubation was successfully avoided in 54% of ARDS patients [23].

Our data represent the largest retrospective cohort studying the outcomes of NIPPV in ARDS to date. The analysis indicated for the first time the lack of statistically significant differences in the adjusted all-cause in-hospital mortality rate and LOS between NIPPV failure and initial endotracheal intubation. Our results also emphasized that if NIPPV is successful, it is associated with significantly lower all-cause in-hospital mortality rates and LOS compared with initial intubation, a finding that is consistent with the previously published literature [7, 24]. Therefore, an initial trial of NIPPV may be considered in ARDS since NIPPV could, on the one hand, be potentially beneficial and, on the other hand, does not seem to result in worse outcome if it fails compared with initial intubation. A large prospective trial is warranted to confirm these findings.

Additionally, our analysis showed that sepsis, pneumonia, and chronic liver disease are independently associated with higher odds for NIPPV failure. In addition to other risk factors such as higher nonpulmonary Sequential Organ Failure Assessment (SOFA) score and respiratory rate [8], these conditions could be used to stratify patients to the most suitable ventilator modality, i.e., NIPPV vs. IMV. However, further prospective research is required to validate this finding.

### 4.1. Strengths and Limitations

Our findings are strengthened by several factors. HCUP-NIS provides a nationally representative sample, making our data the closest approximation of national trends [11]. Additionally, our dataset rigorously adheres to the NIS methodological standards [19], and our algorithm for identifying ARDS records has led to an enormous statistical power as it captured a significantly larger number of observations compared with all previously published studies. Also, the Berlin definition of ARDS was strictly followed in the dataset included [20], and all records with ARF that was attributed to ACPE, those who did not receive any positive pressure ventilation, or those who were intubated before admission, i.e., when the diagnosis of ARDS might not be clearly linked to ARDS at the time of intubation, were excluded.

Of note, our findings are limited by the inherent biases of retrospective analyses involving large administrative databases, including confounding effects and coding errors [25]. Several factors, such as variations in billing, physician documentation, and practices influencing accurate assignments of ICD-10 codes, may have led to inaccuracies in estimating the diagnosis of certain comorbidities and complications, making disease misclassification a possibility [14]. To improve the accuracy, we utilized ICD codes that were validated in previous studies, and we captured records with combined inclusion criteria of having ARDS as a principal diagnosis and the provision of positive pressure ventilation as the procedure of interest. Hence, we obtained the most accurate representative sample of ARDS.

In addition, due to restrictions in data elements provided by HCUP-NIS, some variables that were thought to be important in determining ARDS outcomes, such as disease severity, IMV settings, NIPPV interface, duration of the evolving complications, and SOFA score, were not obtainable [18]. Additionally, the duration of the assigned procedure used, whether IMV or NIPPV, was not obtainable due to the nature of the database [11]; it is thought to be an important factor in reflecting which procedure predominantly affected the studied outcome.

## 5. Conclusions

Our analysis shows that NIPPV success in ARDS carries significantly lower mortality rates and shorter LOS compared with IMV. If NIPPV fails, it carries no significant difference in hospital mortality or LOS compared with patients who were initially intubated. Therefore, an initial trial of NIPPV may be considered in ARDS since NIPPV could, on the one hand, be potentially beneficial and, on the other hand, does not seem to result in worse outcome if it fails compared with initial intubation. Also, our data are the first to indicate that sepsis, pneumonia, and chronic liver disease are strong predictors of NIPPV failure; therefore, they should be used to stratify patients to the most suitable ventilation modality. Further studies are required to validate our findings and to establish the causes of these observations.

## Figures and Tables

**Table 1:** Patient and hospital characteristics, ARDS etiologies, comorbidities, and complications, by the study group.

	IMV (*N* = 14,550)		NIPPV (*N* = 6,835)		NIPPV success (*N* = 5,480)		NIPPV failure (*N* = 1,355)	
	%	95% CI	%	95% CI	%	95% CI	%	95% CI
Patient-level characteristics								
Age at admission (years), mean	56.3	[55.7, 57]	61.5	[60.6, 62.4]	62.2	[61.1, 63.2]	58.8	[56.9, 60.6]
Sex, %								
Male (*n* = 2,149)	52.1	[50.2, 53.9]	46.4	[43.7, 49.1]	45.3	[42.3, 48.4]	50.7	[44.8, 56.7]
Female (*n* = 2,127)	47.9	[46.1, 49.8]	53.6	[50.9, 56.3]	54.7	[51.6, 57.7]	49.3	[43.3, 55.2]
Race (uniform)								
White (*n* = 2,471)	63.3	[61.2, 65.3]	54.9	[51.7, 58.1]	53.4	[50.0, 56.7]	61.2	[54.5, 67.6]
Black (*n* = 815)	18.3	[16.7, 20.1]	23.4	[20.8, 26.2]	25	[22.2, 28.0]	16.7	[12.5, 21.9]
Hispanic (*n* = 492)	11.5	[10.1, 13.0]	13.2	[11.2, 15.6]	13.2	[11.0, 15.7]	13.6	[9.7, 18.6]
Asian/Pacific Islander (*n* = 142)	3.2	[2.6, 3.9]	4.2	[3.2, 5.5]	4.4	[3.3, 6.0]	3.1	[1.6, 6.1]
Native American (*n* = 25)	0.7	[0.4, 1.2]	0.5	[0.2, 1.0]	0.4	[0.1, 1.0]	0.8	[0.2, 3.1]
Other (*n* = 135)	3	[2.4, 3.8]	3.9	[2.9, 5.2]	3.7	[2.7, 5.0]	4.7	[2.7, 7.9]
Location/teaching status of hospital, %								
Rural (*n* = 263)	5.6	[4.9, 6.4]	7.3	[5.9, 9.0]	6.5	[5.1, 8.3]	10.7	[7.6, 14.9]
Urban, nonteaching (*n* = 931)	21	[19.4, 22.6]	23.4	[20.7, 26.4]	23.1	[20.2, 26.3]	24.7	[19.7, 30.5]
Urban, teaching (*n* = 3,083)	73.4	[71.6, 75.1]	69.3	[66.1, 72.3]	70.4	[67.0, 73.6]	64.6	[58.3, 70.4]

Hospital-level characteristics								
Region of hospital, %								
Northeast (*n* = 826)	17.4	[15.8, 19.2]	23.3	[20.3, 26.7]	24.5	[21.0, 28.2]	18.8	[14.3, 24.4]
Midwest (*n* = 846)	22.4	[20.3, 24.7]	14.2	[11.7, 17.1]	13.2	[10.7, 16.3]	18.1	[13.7, 23.4]
South (*n* = 1,724)	39.3	[37.1, 41.6]	42.4	[39.0, 46.0]	42.4	[38.7, 46.2]	42.4	[36.0, 49.2]
West (*n* = 881)	20.9	[19.0, 22.8]	20	[17.5, 22.8]	19.9	[17.1, 23.0]	20.7	[16.0, 26.3]

ARDS etiologies								
Sepsis, %, (*n* = 1,885)	50.8	[48.9, 52.6]	29.8	[27.4, 32.4]	21.4	[19.0, 23.9]	64.2	[58.3, 69.7]
Pneumonia, %, (*n* = 1,654)	42.3	[40.5, 44.2]	30.9	[28.5, 33.4]	24.6	[22.2, 27.3]	56.1	[50.1, 61.9]
Acute pancreatitis, %, (*n* = 138)	3.7	[3.1, 4.5]	2.1	[1.5, 3.0]	2	[1.3, 3.0]	2.6	[1.2, 5.3]
TRALI, %, (*n* = 16)	0.4	[0.3, 0.8]	0.2	[0.1, 0.7]	0.1	[0.0, 0.6]	0.7	[0.2, 2.9]
Trauma, %, (*n* = 392)	11.2	[9.9, 12.6]	4.9	[3.9, 6.2]	4.8	[3.7, 6.3]	5.2	[3.1, 8.5]

Comorbidities								
COPD, %, (*n* = 1,016)	20.1	[18.7, 21.7]	31.5	[29.0, 34.0]	31	[28.3, 33.9]	33.2	[27.7, 39.2]
Chronic CHF, %, (*n* = 441)	9.2	[8.2, 10.3]	12.7	[11.0, 14.6]	13.9	[11.9, 16.0]	8.1	[5.3, 12.3]
Chronic IHD, %, (*n* = 935)	19.9	[18.3, 21.5]	26.1	[23.8, 28.5]	28.4	[25.8, 31.1]	17	[13.1, 21.7]
CKD, %, (*n* = 1,116)	21.3	[19.8, 22.8]	36.4	[33.8, 39.0]	39.1	[36.2, 42.0]	25.5	[20.7, 31.0]
Chronic liver disease, %, (*n* = 394)	10.4	[9.4, 11.6]	6.6	[5.4, 8.0]	4.7	[3.6, 6.0]	14.4	[10.7, 19.0]
Malignant neoplasms, %, (*n* = 402)	9.2	[8.2, 10.4]	9.8	[8.3, 11.5]	9.6	[8.0, 11.5]	10.7	[7.6, 14.9]
Dementia, %, (*n* = 172)	3.4	[2.8, 4.1]	5.3	[4.2, 6.7]	5.6	[4.3, 7.2]	4.4	[2.5, 7.6]
Hypertension, %, (*n* = 2,695)	57.9	[55.9, 59.8]	74	[71.5, 76.2]	77.5	[74.9, 79.9]	59.8	[53.5, 65.8]
DM with/without complications, %, (*n* = 1,413)	30.1	[28.3, 31.9]	39.4	[36.8, 42.0]	41.9	[39.0, 44.8]	29.2	[24.2, 34.7]
Obesity, %, (*n* = 847)	18.2	[16.8, 19.7]	23.1	[20.9, 25.5]	23.2	[20.7, 25.8]	22.9	[18.1, 28.4]
Smoking, %, (*n* = 795)	18.9	[17.4, 20.4]	18	[16.0, 20.2]	17.6	[15.3, 20.2]	19.6	[15.3, 24.7]

Complications								
Shock state, (*n* = 1,378)	40.8	[38.9, 42.7]	14	[12.2, 15.9]	6.8	[5.5, 8.5]	42.8	[37.1, 48.7]
Acute kidney failure, %, (*n* = 1,890)	49.6	[47.7, 51.4]	32.8	[30.3, 35.4]	27.3	[24.7, 30.0]	55	[48.9, 60.9]
Acute liver failure, %, (*n* = 218)	6.7	[5.8, 7.7]	1.8	[1.2, 2.6]	0.8	[0.4, 1.6]	5.5	[3.4, 8.9]
DIC and coagulopathy, %, (*n* = 101)	2.7	[2.2, 3.4]	1.5	[1.0, 2.4]	0.5	[0.2, 1.1]	5.9	[3.6, 9.6]

*n*: actual number of observations; *N*: population-weighted sample size; CI: confidence interval; ARDS: acute respiratory distress syndrome; IMV: invasive mechanical ventilation; NIPPV: noninvasive positive pressure ventilation; TRALI: transfusion-related acute lung injury; COPD: chronic obstructive pulmonary disease; CHF: congestive heart failure; IHD: ischemic heart disease; CKD: chronic kidney disease; DM: diabetes mellitus; DIC: disseminated intravascular coagulopathy.

**Table 2:** All-cause patient in-hospital mortality rate (%) and length of stay (days), by the study group.

	IMV (*N* = 14,550)		NIPPV (*N* = 6,835)		NIPPV success (*N* = 5,480)		NIPPV failure (*N* = 1,355)	
	%, mean	95% CI	%, mean	95% CI	%, mean	95% CI	%, mean	95% CI
Died during hospitalization, %, (*n* = 858)	25.1	[23.5, 26.9]	9.3	[7.8, 11.0]	4.9	[3.8, 6.4]	26.9	[21.8, 32.8]
Length of stay (days), mean	15.6	[15, 16.3]	8.8	[8.2, 9.4]	7	[6.6, 7.5]	16	[14, 18]

*N*: population-weighted sample size; *n*: actual number of observations; CI: confidence interval; IMV: invasive mechanical ventilation, NIPPV: noninvasive positive pressure ventilation.

## Data Availability

The data used to support the findings of this study are available from the corresponding author upon request.

## Supplementary Materials

Supplementary Materials Table S1: the International Classification of Diseases, Tenth Revision, Clinical Modification and Procedure Coding System (ICD-10 CM/PCS) Codes that were used to identify the dataset records. Table S2: the predicting factors for NIPPV failure. Table S3: the predicting factors for log-transformed length of stay. Table S4: the predicting factors for all-cause in-hospital mortality.
