# Supplementary material for: Outcomes of Noninvasive Positive Pressure Ventilation in Acute Respiratory Distress Syndrome and Their Predictors: A National Cohort
Source: Crit Care Res Pract. 2019 Sep 18;2019:8106145. doi: 10.1155/2019/8106145 (PMC6766679; doi:10.1155/2019/8106145)
Supplement: Supplementary Materials — Table S1: the International Classification of Diseases, Tenth Revision, Clinical Modification and Procedure Coding System (ICD-10 CM/PCS) Codes that were used to identify the dataset records. Table S2: the predicting factors for NIPPV failure. Table S3: the predicting factors for log-transformed length of stay. Table S4: the predicting factors for all-cause in-hospital mortality. [file 8106145.f1.docx]

**Online Supplement**

**Outcomes of Non-invasive Positive Pressure Ventilation in Acute Respiratory Distress Syndrome and Their Predictors: A National Cohort**

Ahmed Taha MD^1^, Eneko Larumbe-Zabala PhD^2^, Ashraf Abugroun MD^3^, Assad Mohammedzein MD ^1^, M Tarek Naguib MD ^1^, Manish Patel MD ^1,4^

^1^Department of Internal Medicine, Texas Tech University Health Sciences Center, Amarillo, TX, USA

^2^Clinical Research Institute, Texas Tech University Health Sciences Center, Lubbock, TX, USA

^3^Department of Internal Medicine, Advocate Illinois Masonic Medical Centre, Chicago, IL, USA

^4^Department of Internal Medicine, Division of Pulmonary and Critical Care Medicine, Texas Tech University Health Sciences Center, Amarillo, TX, USA

**ORCID:** 0000-0002-8243-2936 (A.T.), 0000-0002-8949-0602 (E.L.Z.), 0000-0003-3552-5635 (M.T.N), 0000-0002-7195-7358 (M.P).

**Table S1. The International Classification of Diseases, Tenth Revision, Clinical Modification and Procedure Coding System (ICD-10 CM/PCS) Codes That Were Used To Identify the Dataset Records.**

| **Procedure** | **ICD-10 PCS code** |
| --- | --- |
|  |  |
| IMV | 0BH17EZ, 0BH18EZ, 5A1935Z, 5A0945Z, 5A0955Z |
| NIPPV | 5A09357, 5A09457, 5A09458, 5A09358, 5A09557, 5A09558 |
|  |  |
| **Diagnosis** | **ICD-10 CM code** |
|  |  |
| ARDS | J80, J810 |
| ACPE | I5021, I5023, I5033, I5031, I5041, I5043 |
| DNR | Z66 |
| **ARDS Etiologies** | |
| Sepsis | A41**, R65** |
| Pneumonia | J12**, J13, J14, J15**, J16**, J17, J18** |
| acute pancreatitis | K85** |
| TRALI | J9584 |
| Trauma | S00**-S99**, T07, T14** |
| Near-drowning | T75.1 |
| **Comorbidities** | |
| COPD | J41*, J42, J43*, J44*, J47* |
| Chronic CHF | I501, I5020, I5022, I5030, I5032, I5040, I5042, I509 |
| Chronic IHD | I25** |
| CKD | N18* |
| ESRD | 5A1D70Z, 5A1D80Z, 5A1D90Z, 3E1M39Z |
| Chronic liver disease | K70**, K71**, K721*, K73**, K74**, K75**, K76**, K77 |
| Malignant neoplasms | C00**-C26**, C30**-C41**, C43**-C58**, C60**-C80**,  C81**-C96** |
| Dementia | F01**, F02**, F03**, G30*, G31*, G32** |
| Hypertension | I10, I11*, I12*, I13**, I15* |
| DM w/wo complication | E10**, E11** |
| Obesity | E66** |
| **Complications** | |
| Shock state | R57**, R65.21, T79.4, T78.2, T78.0, T80.5, T75.4, A48.3, T81.1, 3E030XZ, 3E040XZ |
| Acute kidney failure | N 17** |
| Acute liver failure | K720*, K712 |
| DIC and coagulopathy | D65 |

Definition of abbreviations: *: Numbers 0, 1-9; ARDS: Acute respiratory distress syndrome; IMV: Invasive mechanical ventilation; NIPPV: Noninvasive positive pressure ventilation; ACPE: Acute cardiogenic pulmonary edema; DNR: Do not resuscitate; TRALI: Transfusion-related acute lung injury; COPD: Chronic obstructive pulmonary disease; CHF: Congestive heart failure; IHD: Ischemic heart disease; CKD: Chronic kidney disease; ESRD: End stage renal disease; DM: Diabetes mellitus; DIC: Disseminated intravascular coagulopathy.

**Table S2. Predicting Factors for NIPPV Failure.**

|  | **OR** | **95% CI** | **p-value** | **aOR** | **95% CI** | **p-value** |
| --- | --- | --- | --- | --- | --- | --- |
|  |  |  |  |  |  |  |
| Sepsis | 6.96 | 5.23–9.26 | <0.001 | 4.47 | 3.24–6.17 | **<0.001** |
| Pneumonia | 4.21 | 3.19–5.55 | <0.001 | 2.65 | 1.94–3.62 | <0.001 |
| Acute pancreatitis | 1.25 | 0.52–3.01 | 0.510 |  |  |  |
| TRALI | 7.17 | 0.64–80.05 | 1.600 |  |  |  |
| Trauma | 1.09 | 0.6–1.98 | 0.280 |  |  |  |
| COPD | 1.15 | 0.86–1.54 | 0.970 |  |  |  |
| Chronic CHF | 0.53 | 0.33–0.86 | -2.550 |  |  |  |
| Chronic IHD | 0.54 | 0.39–0.75 | <0.001 | 0.78 | 0.53–1.14 | 0.201 |
| CKD | 0.52 | 0.39–0.7 | <0.001 | 1.08 | 0.76–1.54 | 0.676 |
| CLD | 3.29 | 2.12–5.1 | <0.001 | 2.23 | 1.33–3.75 | **0.003** |
| Neoplasms | 1.20 | 0.77–1.85 | 0.800 |  |  |  |
| Dementia | 0.80 | 0.42–1.53 | -0.680 |  |  |  |
| Hypertension | 0.42 | 0.31–0.57 | <0.001 | 0.59 | 0.41–0.85 | **0.004** |
| Diabetes Mellitus | 0.57 | 0.43–0.76 | <0.001 | 0.73 | 0.53–1.01 | 0.058 |
| Obesity | 0.98 | 0.71–1.36 | -0.110 |  |  |  |
| Smoking | 1.12 | 0.79–1.59 | 0.650 |  |  |  |

Definition of abbreviations: NIPPV: Noninvasive positive pressure ventilation; OR: Unadjusted odds ratios (calculated using simple logistic regression); CI: Confidence interval; aOR: Adjusted odds ratios (calculated using multiple logistic regression); TRALI: Transfusion-related acute lung injury; COPD: Chronic obstructive pulmonary disease; CHF: Congestive heart failure; IHD: Ischemic heart disease; CKD: Chronic kidney disease; CLD: Chronic liver disease.

**Table S3. Predicting Factors for Log-transformed Length of Stay.**

|  | **Coef** | **95% CI** | **p-value** | **Adj Coef** | **95% CI** | **p-value** |
| --- | --- | --- | --- | --- | --- | --- |
| Group  (vs. NIPPV success) |  |  |  |  |  |  |
| IMV | 0.740 | 0.668 – 0.807 | <0.001 | 0.527 | 0.459 – 0.595 | **<0.001** |
| NIPPV Failure | 0.810 | 0.686 – 0.931 | <0.001 | 0.521 | 0.398 – 0.645 | **<0.001** |
| Sepsis | 0.620 | 0.56 – 0.682 | <0.001 | 0.369 | 0.304 – 0.435 | **<0.001** |
| Pneumonia | 0.530 | 0.47 – 0.592 | <0.001 | 0.332 | 0.271 – 0.393 | **<0.001** |
| Acute pancreatitis | 0.540 | 0.392 – 0.693 | <0.001 | 0.427 | 0.27 – 0.585 | **<0.001** |
| TRALI | 0.330 | -0.101 – 0.767 | 0.132 |  |  |  |
| Trauma | 0.330 | 0.219 – 0.435 | <0.001 | 0.314 | 0.213 – 0.414 | **<0.001** |
| COPD | -0.080 | -0.151 – -0.004 | 0.039 | 0.025 | -0.044 – 0.093 | 0.477 |
| Chronic CHF | -0.050 | -0.158 – 0.051 | 0.317 |  |  |  |
| Chronic IHD | -0.220 | -0.293 – -0.149 | <0.001 | -0.045 | -0.113 – 0.023 | 0.191 |
| CKD | -0.250 | -0.321 – -0.182 | <0.001 | -0.059 | -0.126 – 0.008 | 0.085 |
| CLD | 0.310 | 0.208 – 0.415 | <0.001 | 0.173 | 0.07 – 0.276 | **0.001** |
| Neoplasms | 0.200 | 0.097 – 0.312 | <0.001 | 0.139 | 0.036 – 0.243 | **0.008** |
| Dementia | 0.040 | -0.109 – 0.184 | 0.618 |  |  |  |
| Hypertension | -0.190 | -0.265 – -0.121 | <0.001 | 0.034 | -0.037 – 0.105 | 0.350 |
| Diabetes Mellitus | -0.130 | -0.199 – -0.062 | <0.001 | 0.007 | -0.056 – 0.069 | 0.831 |
| Obesity | 0.040 | -0.037 – 0.117 | 0.311 |  |  |  |
| Smoking | -0.100 | -0.179 – -0.021 | 0.014 | -0.119 | -0.193 – -0.044 | **0.002** |

Definition of abbreviations: Coef: Unadjusted coefficients (calculated using simple linear regression); Adj Coef: Adjusted coefficients (calculated using multiple linear regression); CI: Confidence interval; NIPPV: Noninvasive positive pressure ventilation; IMV: Invasive mechanical ventilation; TRALI: Transfusion-related acute lung injury; COPD: Chronic obstructive pulmonary disease; CHF: Congestive heart failure; IHD: Ischemic heart disease; CKD: Chronic kidney disease; CLD: Chronic liver disease.

**Table S4. Predicting Factors for All-cause In-hospital Mortality.**

|  | **OR** | **95% CI** | **p-value** | **aOR** | **95% CI** | **p-value** |
| --- | --- | --- | --- | --- | --- | --- |
| Group (vs. NIPPV success) |  |  |  |  |  |  |
| IMV | 6.48 | 4.88–8.61 | <0.001 | 5.30 | 3.96–7.11 | **<0.001** |
| NIPPV Failure | 7.11 | 4.83–10.48 | <0.001 | 5.43 | 3.61–8.17 | **<0.001** |
| Sepsis | 2.65 | 2.27–3.1 | <0.001 | 2.02 | 1.71–2.4 | **<0.001** |
| Pneumonia | 1.24 | 1.06–1.44 | 0.007 | 0.82 | 0.69–0.97 | **0.023** |
| Acute pancreatitis | 1.01 | 0.66–1.55 | 0.949 |  |  |  |
| TRALI | 0.92 | 0.26–3.2 | 0.894 |  |  |  |
| Trauma | 1.19 | 0.95–1.5 | 0.134 |  |  |  |
| COPD | 0.85 | 0.7–1.02 | 0.087 |  |  |  |
| Chronic CHF | 0.88 | 0.69–1.14 | 0.336 |  |  |  |
| Chronic IHD | 0.96 | 0.8–1.15 | 0.664 |  |  |  |
| CKD | 0.67 | 0.56–0.8 | <0.001 | 0.97 | 0.79–1.19 | **<0.001** |
| CLD | 2.00 | 1.59–2.51 | <0.001 | 1.59 | 1.25–2.04 | **<0.001** |
| Neoplasms | 2.41 | 1.92–3.01 | <0.001 | 2.27 | 1.78–2.89 | **<0.001** |
| Dementia | 1.14 | 0.79–1.63 | 0.495 |  |  |  |
| Hypertension | 0.58 | 0.49–0.68 | <0.001 | 0.82 | 0.68–0.99 | **0.037** |
| Diabetes Mellitus | 0.72 | 0.61–0.85 | <0.001 | 0.91 | 0.76–1.1 | 0.337 |
| Obesity | 0.59 | 0.48–0.73 | <0.001 | 0.69 | 0.55–0.86 | **0.001** |
| Smoking | 0.54 | 0.43–0.67 | <0.001 | 0.52 | 0.41–0.65 | **<0.001** |

Definition of abbreviations: OR: Unadjusted odds ratios (calculated using simple logistic regression; aOR: Adjusted odds ratios (calculated using multiple logistic regression); CI: Confidence interval; NIPPV: Noninvasive positive pressure ventilation; IMV: Invasive mechanical ventilation; TRALI: Transfusion-related acute lung injury; COPD: Chronic obstructive pulmonary disease; CHF: Congestive heart failure; IHD: Ischemic heart disease; CKD: Chronic kidney disease; CLD: Chronic liver disease.
